# Future research and collaboration: the “SINERGIE” project on HCV (*South Italian Network for Rational Guidelines and International Epidemiology*)

**DOI:** 10.1186/1471-2334-12-S2-S9

**Published:** 2012-11-12

**Authors:** C Torti, M Zazzi, L Abenavoli, F Trapasso, F Cesario, D Corigliano, L Cosco, C Costa, RL Curia, M De Rosa, G Foti, C Giraldi, R Leone, MC Liberto, D Lucchino, N Marascio, R Masciari, G Matera, V Pisani, N Serrao, L Surace, E Zicca, F Castelli, M Ciccozzi, M Puoti, A Focà

**Affiliations:** 1 Unit of Infectious Diseases, University “Magna Graecia”, Catanzaro, Italy; 2 Institute of Microbiology, University of Siena, Siena, Italy; 3 Department of Health Sciences, University “Magna Graecia”, Catanzaro, Italy; 4 Unit of Medical Genetics, University “Magna Graecia”, Catanzaro, Italy; 5 Unit of Infectious Diseases, “Annunziata” Hospital, Cosenza, Italy; 6 Unit of Infectious Diseases, Ospedale "G. Jazzolino", Vibo Valentia, Italy; 7 Unit of Infectious Diseases, “Pugliese-Ciaccio” Hospital, Catanzaro, Italy; 8 Department of Prevention, Calabria Region Health Authority, Catanzaro, Italy; 9 Unit of Microbiology and Virology, “Bianchi-Melacrino-Morelli” Hospital, Reggio Calabria, Italy; 10 Unit of Infectious Diseases, “Bianchi-Melacrino-Morelli” Hospital, Reggio Calabria, Italy; 11 Unit of Microbiology and Virology, “Annunziata” Hospital, Cosenza, Italy; 12 Unit of Microbiology and Virology, General Hospital, Lamezia Terme, Italy; 13 Unit of Clinical Microbiology, “Magna Graecia” University, Catanzaro, Italy; 14 Unit of Infectious Diseases, Lamezia Terme General Hospital, Lamezia Terme, Italy; 15 Unit of Virology and Microbiology, “Pugliese-Ciaccio” Hospital, Catanzaro, Italy; 16 Unit of Infectious Diseases, ASP Crotone, Crotone, Italy; 17 International Health Department, Lamezia Terme General Hospital, Lamezia Terme, Italy; 18 Infectious and Tropical Diseases Department, University of Brescia, Brescia, Italy; 19 National Institutes of Health, Rome, Italy; 20 Department of Infectious Diseases, “Niguarda-Cà Granda” Hospital, Milan, Italy

## Abstract

The SINERGIE (South Italian Network for Rational Guidelines and International Epidemiology) project is intended to set up a collaborative network comprising virologists, clinicians and public health officials dealing with patients affected by HCV disease in the Calabria Region. A prospective observational data-base of HCV infection will be developed and used for studies on HCV natural history, response to treatment, pharmaco-economics, disease complications, and HCV epidemiology (including phylogenetic analysis). With this approach, we aim at improving the identification and care of patients, focusing on upcoming research questions. The final objective is to assist in improving care delivery and inform Public Health Authorities on how to optimize resource allocation in this area.

## Mission, general objectives, and framework

The mission of the SINERGIE Study Group is to implement evidence-based research on Hepatitis C virus (HCV) into practice. We recognize that evidence from randomized clinical trials (RCT) is not fully informative in the clinical practice because patients are selected for inclusion into RCT based on strict criteria and study design is often not transferable to the diverse circumstances encountered in clinical practice. In fact, patients treated in clinical practice may be affected by bio-psycho-social co-morbidities that can affect treatment acceptability, retention into care, and outcome. Therefore, there is a need to ascertain treatment effectiveness in clinical practice, improve treatment algorithms based on clinical needs, and study their cost-effectiveness through pharmaco-economical analyses. Also, we will try to understand the transferability and the clinical outcomes of existing guidelines for treatment of HCV to guide future policy. In conclusion, we wish to improve the identification and care of patients infected with HCV in the Calabria Region (South Italy), focusing on upcoming research questions that will most advance knowledge in this area and assist in improving care delivery and optimizing resource allocation.

With this objective in mind, we have put together experts from different fields, following a patient-centered approach. In this framework, virologists, infectious disease specialists, gastroenterologists, and public health officials will be called to perform a coordinated effort to adapt the current international guidelines to the specificities and resources available to the Calabria Region’s Health System. Periodical audits will also be conducted and discussed during the annual meetings of the SINERGIE Study Group.

To support our mission, we have developed three overarching goals: (*1*) *Better disease identification*, (*2*) *Better disease management*, and (3) *Improved access and equity.* Goal 1 recognizes that the benefit of better disease management depends first and foremost on identifying infected individuals and linking them to care. Through an improvement in the screening policy of the general population, we will obtain more reliable estimates of the HCV epidemics in order to provide public health officials with relevant information on the status and evolution of the epidemics as a basis for a targeted prevention policy. Phylogenetic analysis will help to understand HCV transmission routes within our population. Goal 2 acknowledges the need for patient-centered, comprehensive, coordinated care and treatment that not only addresses the viral infection but also the social environment, mental and physical complications that affect patients’ outcome. This will take advantage from involvement of specialist clinicians with complementary knowledge, skills and abilities. Goal 3 reaches out to infected patients at highest risk for impaired access, including migrants, the homeless, rural residents, and those with mental health and substance use disorders.

In achieving these goals, we hope to promote a more general understanding of implementation science, contribute to theory development, incorporating the public health assistance philosophy guaranteed by the Italian legislation, and diffusion of innovation frameworks. We will propose to extend this program to other Regions throughout Italy.

## Specific research questions and methods

**Figure 1:**
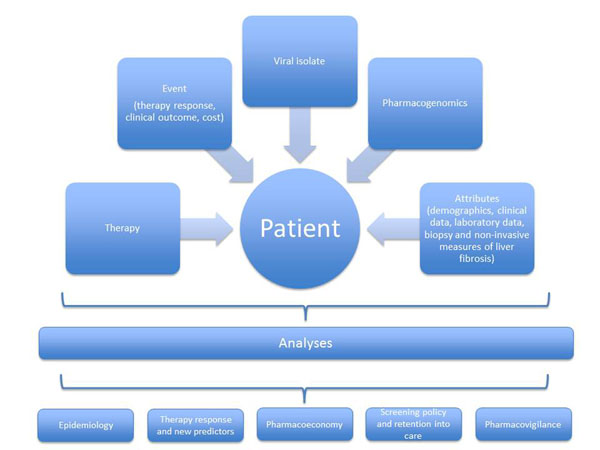
Flow of interventions and objectives of the SINERGIE study

We will conduct a multicenter, prospective, observational study. We recognize that the ability of clinicians to treat HCV patients appropriately will largely depend on a better prediction of the clinical response from a global perspective. In other terms, several variables need to be studied further and integrated into algorithms subjected to clinical validation. The integration of clinical, virological and epidemiological data of the HCV patients included in the SINERGIE database is expected to provide a convenient platform for investigating virus-host interactions in the context of anti-HCV treatment. This can result in models to be applied as a treatment decision support system. Furthermore, the data collected will be used for the molecular monitoring of HCV epidemiology. The general plan of the study (including types of variables –i.e., attributes- recorded) is illustrated in Figure 1.

### A. Clinical and metabolic attributes

Amongst the open questions on the treatment of chronic HCV infection, the following merit to be answered: (i) What is the actual impact of metabolic co-morbidities and/or alcohol abuse on the efficacy of antiviral therapy [1]?; (ii) What is the socio-demographic profile of the HCV population and how this impact on access to treatment and acceptability, retention into care, and treatment outcome [2]?

To try to answer these questions, the project aims at establishing a database of variables that can be analyzed for their association with or prediction of HCV treatment outcome. In particular: age; gender (dichotomized as male, female, and transgender); race/ehnicity (for the analysis, race/ethnicity will be categorized into four categories: white, black, Hispanic, and other/unknown); previous or current substance abuse and types of substances used (substance abuse will be coded as present if an ICD-9 code for substance abuse/dependence is present); previous or current alcohol abuse (the diagnosis of alcoholism will be made based on either the DSM-IIIR or the DSM-IV criteria, or the judgment of the physician for alcohol abuse, based on the history of excessive and persistent alcohol consumption - >20 g ethanol/day for women and >40 g ethanol/day for men, for almost 10 years - along with compatible physical examination and laboratory test abnormalities); HIV positivity (HIV will be coded as present for a positive antibody test confirmed by a western blot or positive HIV RNA); HBsAg positivity; current or previous sexually transmitted disease; stage of liver disease (in particular, end-stage of liver disease, the complications of cirrhosis, such as ascites, hepato-renal syndrome, variceal bleeding, hepatic encephalopathy, spontaneous bacterial peritonitis, will be collected); anthropometric parameters (body weight, height, waist and hip circumferences, waist/hip ratio and body mass index, will be evaluated and collected); biochemical parameters (such as liver markers for intracellular damage, parameters related to metabolic syndrome, will be collected). Ultrasonographic evaluation of the liver parenchyma, portal hemodynamic and spleen status, transient elastography and biochemical non invasive measures will also be monitored and reported at least in a sub-set of patients where these tools are available.

### B. Pharmacogenomic attributes

Recently, different interleukins have been associated with HCV natural history, responses to pegIFN/RBV, and spontaneous clearance of acute HCV infection. In particular, IL28B single nucleotide polymorphisms are associated with spontaneous and treatment-induced clearance of HCV, so treatment guidelines recommend to perform a pharmacogenomic screening before prescribing HCV treatment [3]. However, since other genes may be implicated [4], it is important to conduct pharmacogenomic studies aimed at validating inclusion of interleukin genes polymorphisms into diagnostic and therapeutic algorithms of HCV infection.

### C. Virological attributes and phylogenetic analysis

Genetic variability is a major hallmark of HCV biology. Currently available HCV sequences can be grouped into 6 genotypes, each further sub-divided into several subtypes [5]. The clinical impact of HCV variability has been known since interferon (IFN) based anti-HCV therapy has been introduced in the clinic. Indeed, natural susceptibility to IFN and hence to the standard of care pegylated IFN plus ribavirin (pegIFN/RBV) varies significantly with different HCV genotypes [6]. In particular, HCV genotype 1, the most prevalent in Western countries, appears to be much less sensitive to pegIFN/RBV compared to other genotypes. Other genetic predictors of response to pegIFN/RBV include specific aminoacid variants in the core protein (Arg70 and Leu91) [7] and the composition of the short interferon sensitivity-determining region (ISDR) and interferon and ribavirin resistance-determining region (IRRDR) in the NS5A coding sequence [8,9]. This has established routine designation of HCV genotype through molecular assays as a screening test whenever HCV treatment is considered. Subsequently, the relative inability to eradicate HCV genotype 1 infection by pegIFN/RBV has fuelled large investments ultimately resulting in the development and licensing of the first directly acting antivirals (DAA). Availability of the first two HCV NS3/4A protease inhibitors (boceprevir [BVR] and telaprevir [TVR]) has significantly expanded treatment options and increased the rate of sustained virological response (SVR) [10]. However, adding the current HCV NS3/4A protease inhibitors to the standard pegIFN/RBV therapy has also increased treatment complexity and toxicity.

Given the unprecedented number of compounds being developed against different targets in HCV life cycle [11], monitoring HCV variability through HCV genome sequencing in patients candidate to or under treatment could become an integral part of the clinical management of HCV infection [12]. At this time, it is not possible to foresee to what extent HCV genome sequencing will remain a research need or will be commonly used in clinical practice. Recent trials have shown that HCV drug resistance is frequently detected at treatment failure of NS3/4A protease inhibitors based therapy [13]. Specific drug resistance mutations have been found which are preferentially associated with BVR or TVR and with HCV genotype 1a or 1b. Since no trial on DAA-pretreated subjects has been completed, it is difficult to weight the cost-benefit of drug resistance testing after virological treatment failure. Likewise, the role of genotypic testing beyond genotype and subtype assignment before treatment with DAA is still a matter of debate. Limited analysis of data derived from BVR and TVR phase III trials have suggested that treatment failure due to pre-existent naturally resistant HCV strains has been negligible or hardly detectable [14]. However, majority drug resistance mutations for most DAAs have been documented in a small but variable fraction of untreated subjects in several studies [15-18]. Although understanding the dynamics of DAA resistance in vivo is clearly of interest, expert HCV clinicians may well argument that given the low prevalence and impact of natural resistance and the future availability of many and mostly non-cross-resistant DAA classes there is no clinical need for genotypic resistance testing.

The SINERGIE project aims at improving care of HCV infected patients through integration of clinical, epidemiological, virological and biostatistics expertise. Data collected from these domains into a dedicated on-line database will be analyzed and used to derive patient tailored treatment protocols. The individual HCV strain of each patient included in the database will be characterized for genotype (sequencing of the 5’UTR plus partial core region including the aminoacid sites impacting response to pegIFN/RBV), NS3 sequence (detection of natural polymorphisms potentially affecting susceptibility to NS3/4A protease inhibitors) and NS5A sequence (analysis of the ISDR and IRRDR regions). Patients undergoing NS3/4A protease inhibitor therapy will be monitored for development of NS3/4A protease inhibitor resistance according to reference guidelines [19,20]. Along with development and licensing of novel DAA classes, sequence analysis at baseline and at treatment failure will be extended to other HCV genome regions, e. g. the NS5B RNA-dependent RNA-polymerase gene. Upon insertion into the SINERGIE database, HCV sequences will be checked for quality assurance (e. g., detection of stop codons, excessive degeneration, frameshift mutations, uncommon aminoacids), assigned genotype and subtype and processed for extraction of mutations with respect to the reference strain. Mutations detected will be matched to the reference DAA resistance mutation list and a report indicating the inferred susceptibility to available DAA will be automatically generated.

As for the epidemiological objective, storing HCV sequences in the SINERGIE database will allow phylogenetic and phylogeographic studies exploring the relationships among different clusters of specific HCV variants and patient population features such as transmission routes, origin of viral strains, time of acquisition of infection. This information can generate and periodically refresh a dynamic picture of the circulation of the different HCV strains in the area under analysis.

## Competing interests

The authors declare that they have no competing interests related to the contents of this paper.

## Declarations

Publication of this supplement was partly supported by an unrestricted grant provided by Roche. The articles were independently prepared by the authors with no input from Roche. Roche were not involved in selecting the articles for the supplement. The pegylated IFN/pegIFN treatment mentioned in this article is produced by Roche.
